# Efficacy of Mediterranean diet interventions on children and adolescents affected by overweight or obesity: a systematic review and meta-analysis

**DOI:** 10.3389/fnut.2026.1749319

**Published:** 2026-08-26

**Authors:** Iro Rapti, Georgios Markozannes, Ourania Papathanasiou, Antonia Veroni, Christos Zachariades, Xanthippi Tseretopoulou, Eleni P. Kotanidou, Anastasios Serbis, Vasilios Koutras, Evangelos C. Rizos, Evangelia Ntzani

**Affiliations:** 1 Department of Hygiene and Epidemiology, School of Health Sciences, University of Ioannina School of Medicine, Ioannina, Greece; 2 Department of Internal Medicine, Faculty of Medicine, School of Health Sciences, University of Ioannina, Ioannina, Greece; 3 Department of Paediatrics, Royal Aberdeen Children’s Hospital, Aberdeen, United Kingdom; 4 Second Department of Pediatrics, School of Medicine, Faculty of Health Sciences, Aristotle University of Thessaloniki, AHEPA University Hospital, Thessaloniki, Greece; 5 Department of Pediatrics, School of Health Sciences, University of Ioannina School of Medicine, Ioannina, Greece; 6 Department of Early Childhood Education, University of Ioannina School of Education, Ioannina, Greece; 7 Department of Nursing, School of Health Sciences, University of Ioannina, Ioannina, Greece; 8 Center for Evidence Synthesis in Health, Department of Health Services, Policy and Practice, School of Public Health, Brown University, Providence, RI, United States; 9 Institute of Biosciences, University Research Center of loannina, University of Ioannina, Ioannina, Greece

**Keywords:** adolescents, children, Mediterranean diet, obesity, overweight

## Abstract

**Background/objectives:**

Overweight and obesity in children and adolescents is a global health issue with increasing prevalence. The Mediterranean diet (Med diet) is an extensively studied dietary pattern with benefits on various health conditions such as cardiovascular disease, diabetes mellitus and obesity in adult populations but the evidence on children and adolescents remains limited. The aim of this systematic review and meta-analysis is to collect, assess, and synthesize evidence from randomized controlled trials (RCTs) on the efficacy of Med diet on anthropometric and cardiometabolic parameters in children and adolescents with excess body weight.

**Methods:**

Three electronic databases (PubMed, Cochrane Central Register of Controlled Trials, Scopus) and references in relevant studies and systematic reviews were searched. Random-effects meta-analysis models were used to synthesize study effect estimates. Heterogeneity was assessed using the *I*^2^. The quality of evidence was evaluated using the Cochrane Risk of Bias tool v2.0.

**Results:**

Five RCTs involving 330 children and adolescents affected by overweight or obesity were included. Mean study duration was 12 weeks (range: 8–16 weeks). Compared with the control group, Med diet was significantly associated with lower body mass index (BMI) (Standardized mean difference [SMD] = −0.26, 95% confidence intervals [CI] = −0.49 to −0.03, *I*^2^ = 0%) and lower systolic blood pressure (SMD = −0.48, 95% CI = −0.87 to −0.09, *I*^2^ = 45.9%). No statistically significant associations were observed for any other anthropometric or cardiometabolic parameter studied. Regarding risk of bias, all RCTs were judged to have some concerns for each outcome assessed.

**Conclusion:**

This systematic review suggests that Med diet could have a potentially beneficial effect on the reduction of BMI among youths with excess body weight. Nevertheless, further research, with greater methodological rigor, higher participation rates and longer follow-up is needed.

**Systematic Review Registration:**

This systematic review and meta-analysis is registered with PROSPERO (CRD420251143115).

## Introduction

Obesity in children and adolescents is a major health issue with increasing prevalence worldwide (1). In 2022, approximately 390 million children and adolescents, aged 5 to 19 years, were affected by either overweight or obesity. The prevalence of overweight and obesity among children and adolescents has risen considerably from 8% in 1990 to 20% in 2022, while at the same time 160 million children and adolescents (8%) were living with obesity (1). In many cases, obesity during childhood continues during adulthood and is associated with higher risk of development of non-communicable diseases such as cardiovascular disease and diabetes mellitus as well as premature mortality (1–4). Moreover, obesity during childhood and adolescence is associated with adverse psychosocial parameters and impaired quality of life (1, 4). Development and maintenance of obesity involves a complex framework including biological predisposition, socioeconomical and environmental factors (4). The development and provision of effective interventions for children and their families is vitally important for the prevention and treatment of obesity during childhood and its implications for future health.

The Mediterranean diet (Med diet) is one of the most extensively studied dietary patterns, which has gained recognition for its benefits on various health conditions in adult population such as cardiovascular disease, diabetes mellitus and obesity (5, 6). Med diet is characterized by the use of olive oil as the primary dietary fat, abundant consumption of fruits, vegetables, legumes, whole grains, cereals, nuts and fish, moderate intake of milk and dairy products and low intake of red or processed meats and ultra-processed foods (7–9). Even though there is extensive evidence that the Med diet pattern is associated with health benefits as regards non-communicable diseases in adults (5, 10, 11), the literature pertaining to the efficacy of Med diet on children and adolescents is restricted. According to a meta-analysis of 15 randomized controlled trials (RCTs) focusing on the effect of Med diet on anthropometric and obesity-related parameters in children and adolescents regardless of their weight status, Med diet is associated with a small reduction of body mass index and a decrease in the proportion of obesity among children in the intervention group (12). Another recent meta-analysis which included 9 RCTs pertaining to children and adolescents with normal and excess body weight showed an association between Med diet and improved cardiometabolic parameters (13). Moreover, lower adherence to Med diet was associated with increased odds of developing metabolic syndrome among children and adolescents in a meta-analysis of cross-sectional and longitudinal studies (14). However, little is known about the efficacy of Med diet-based interventions among children and adolescents with excess body weight. In previous evidence synthesis, conclusions on the efficacy of Med diet interventions for youths affected by overweight and/or obesity were based either on analyses involving children/adolescents with normal and excess body weight or on subgroups analyses referring to children/adolescents with excess body weight for some anthropometric parameters (e.g., body mass index) (12, 13). The aim of the present systematic review and meta-analysis is to systematically collect, assess and synthesize the evidence of RCTs on the efficacy of Med diet on various anthropometric and cardiometabolic parameters focusing exclusively on children and adolescents affected by overweight and/or obesity.

## Materials and methods

This systematic review was conducted and reported following the Preferred Reporting Items for Systematic Reviews and Meta-Analyses (PRISMA) guidelines (15). This systematic review and meta-analysis is registered with PROSPERO (CRD420251143115).

### Eligibility criteria

Studies were eligible if they met the following criteria based on the population, intervention, comparison, outcome and study design framework. Participants were eligible if: (i) they were children or adolescents, aged 18 years or younger and (ii) they were identified as children or adolescents with overweight or obesity. Interventions were eligible if they referred to any intervention diet explicitly described as related to “Mediterranean diet.” Med diet includes the following key components: (i) high monounsaturated/saturated fat ratio (use of olive oil as a main cooking ingredient and/or consumption of other traditional foods high in monounsaturated fats such as tree nuts), (ii) a high intake of plant-based foods, including fruits, vegetables and legumes, (iii) high consumption of whole grains and cereals, (iv) low consumption of meat and meat products, (v) increased consumption of fish and (vi) moderate consumption of milk and dairy products (7, 9). The control group was any other dietary pattern or usual care. The outcomes of interest were: (i) anthropometric parameters (body mass index [BMI], weight, waist circumference [WC], and body fat) and (ii) cardiometabolic parameters (systolic blood pressure [SBP], diastolic blood pressure [DBP], total cholesterol [TC], high-density lipoprotein [HDL-C], low-density lipoprotein [LDL-C], triglycerides [TRGs], glucose, insulin, glycated hemoglobin [ΗbA1c%], and homeostatic model assessment for insulin resistance [HOMA-IR]). Studies were included if they followed a RCT design.

Studies were excluded if: (i) participants were adults, (ii) no Mediterranean diet arm was available, (iii) the control group was also a Mediterranean diet-related arm, (iv) the intervention arm included other components that were not included in the control arm, and (v) the study did not follow a RCT design. No restrictions were applied regarding sample size or length of intervention.

### Information sources, search strategy, and data extraction

We searched PubMed and the Cochrane Central Register of Controlled Trials (CENTRAL) from inception to September 15th, 2025, and Scopus from inception to June 7th, 2026, following a search strategy based on population, intervention, comparison, outcome and study design framework. The following keywords, index terms and Boolean operators were used for the search: (“Child”[Mesh] OR child* OR “Adolescent”[Mesh] OR adolescen*) AND (“Obesity”[Mesh] OR obes* OR “Overweight”[Mesh] OR overweight) AND (“Diet, Mediterranean”[Mesh] OR “Mediterranean diet” OR “Mediterranean dietary pattern”) AND (“randomized controlled trial” OR “randomised controlled trial” OR “randomized clinical trial” OR “randomised clinical trial” OR “clinical trial”). The search strategy in each database is provided in Supplementary Table S1. In addition, the reference lists of included studies and pertinent systematic reviews and meta-analyses were screened for additional, relevant RCTs. The search strategy had no language restrictions. Firstly, title and abstract screening was done independently by two reviewers followed by full-text screening. A third reviewer was consulted in case of disagreement. Reasons for exclusion of studies were reported during the full-text screening phase. For each eligible study, the following information was recorded by two reviewers independently: first author, year of publication, name of the study (if provided), study’s country of origin, experimental and control arms type and description, outcome of interest, targeted population, number of subjects randomized and number of subjects analyzed in each arm, baseline and follow-up means and standard deviation (SD) or standard error (SE) and change from baseline and SD or SE for each arm at the longest follow-up available and physical activity prescription.

### Risk of bias assessment

The risk of bias was assessed using the Cochrane Collaboration revised tool of Risk of Bias (RoB 2.0) (16) to all the included studies. The tool covers five domains: (i) bias arising from randomization process, (ii) bias due to deviations from intended interventions, (iii) bias due to missing outcome data, (iv) bias in measurement of outcome and (v) bias in the selection of the reported result. Based on the possible risk of bias in each of the five domains, the study was judged as low risk of bias, with some concerns or high risk of bias. Two reviewers assessed the risk of bias independently, while a third reviewer was consulted in case of disagreement.

### Data synthesis and analysis

Data were narratively summarized by outcome. For each outcome, the results were summarized using a random-effects meta-analysis model if at least three trials were available to calculate the summary effect estimate (standardized mean difference [SMD]) along with their 95% confidence intervals (CI). SMDs were calculated using reported follow-up means per group or reported mean change from baseline to follow-up per group. Percentage of variation of the estimates attributable to heterogeneity was calculated using the *I*^2^ statistic. Exploration of small study effects (an indication of publication bias) and meta-regression analyses were not conducted because no more than 10 studies were available for any of the outcomes. As a post-hoc analysis a stratified analysis by type of control group (standard diet, low-fat diet) was performed. To explore the robustness of the summary estimates, sensitivity analyses by excluding one study at a time from the summary estimates were conducted. All analyses were performed with Stata (version 14.1).

### Quality of evidence

The quality of the evidence was appraised using the Grading of Recommendations, Assessment, Development, and Evaluations (GRADE) approach (17). The GRADE approach considers 4 levels of quality (high, moderate, low, and very low). The overall quality of evidence was initially regarded as high (evidence from RCTs) and then downgraded by one or two levels for the following domains: (i) risk of bias (ii) inconsistency, (iii) indirectness of patients, intervention, or comparison, (iv) imprecision and (v) publication bias.

## Results

**Figure 1:**
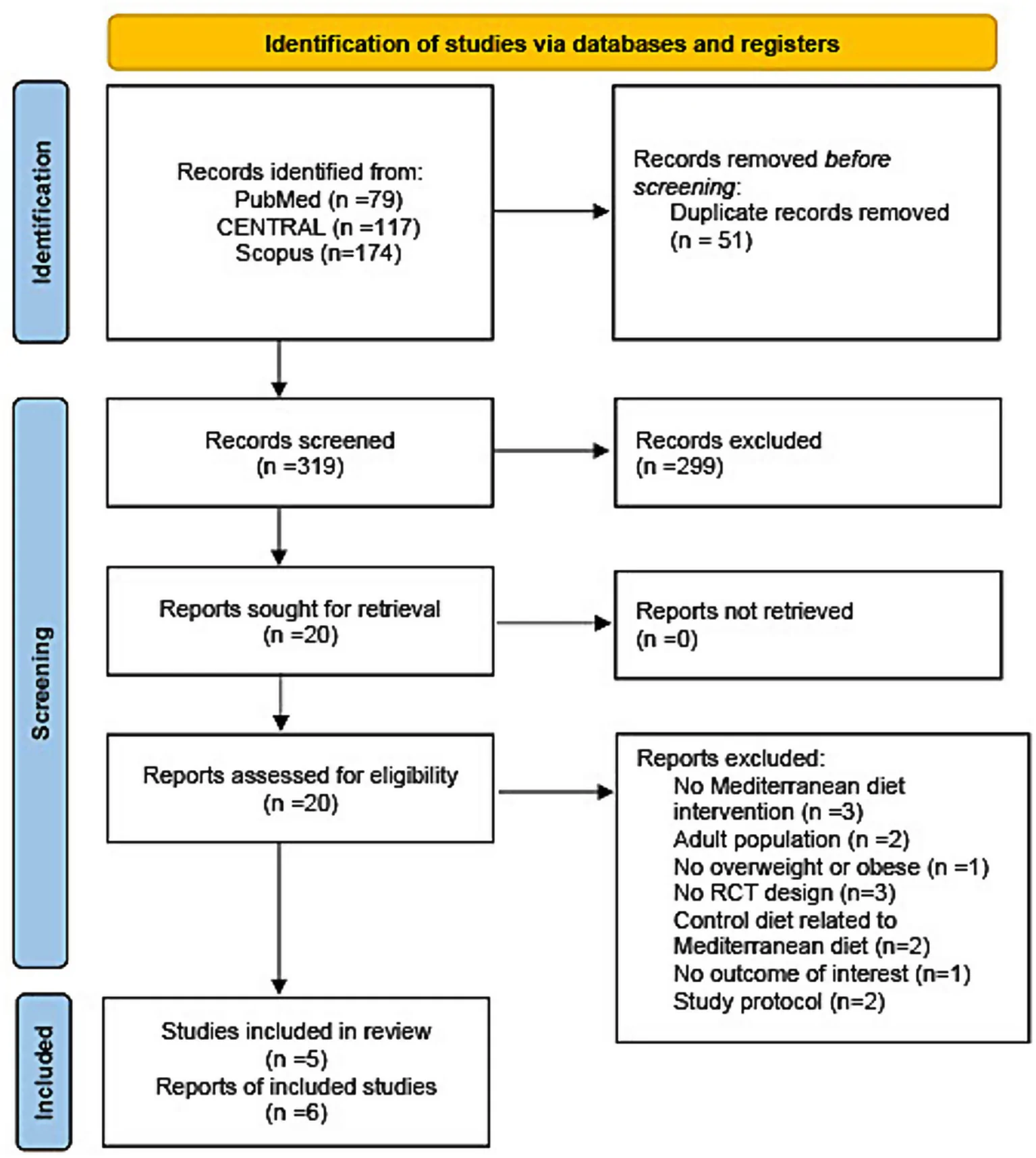
PRISMA flow diagram of study selection.

The search of PubMed, CENTRAL and Scopus provided a total of 370 records. Of the studies identified, 20 (18–37) were eligible for a full-text screening and 6 papers (18, 19, 30–33) reporting on outcomes from 5 RCTs were selected for data extraction and analysis. The PRISMA flow diagram illustrating the selection process is shown in Figure 1.

Overall, we included 5 RCTs (18, 19, 30–33) involving a total of 330 (range: 44–107 per trial) children and adolescents with excess body weight. Mean study duration was 12 weeks (range: 8–16 weeks). Two RCTs were conducted in Turkey (19, 30), one RCT in Iran (18), one in Spain (33) and one in Mexico (31). The intervention groups included a total of 192 participants (range: 22–81 per trial). Two RCTs (19, 30) involved children and adolescents with overweight or obesity and non-alcoholic fatty liver disease, one trial (18) involved exclusively adolescent girls with abdominal obesity and metabolic syndrome, one trial (31) involved children and adolescents with obesity (BMI ≥ 95th percentile) and any metabolic syndrome component and one trial (33) involved children and adolescents with abdominal obesity (WC ≥ 90th percentile according to national data). All Med diet-based interventions included dietary advice based on Med diet recommendations. Dietary counseling was provided by trained dietitians or clinicians through interactive sessions, phone or social media feedback and provision of educational material on Med diet. Counseling sessions involved both children, adolescents and their parents. Three RCTs provided fixed full-day meal plans based on Med diet principles (18, 19, 33). One study involved a moderately hypocaloric Med diet (33). Adherence to Med diet was assessed in 2 RCTs (19, 33) using the KIDMED questionnaires (38). The control group included dietary counseling on either standard diet in 3 trials (18, 31, 33) or low-fat diet in 2 trials (19, 30). All participants, regardless of intervention or control group, were encouraged to maintain or enhance the level of their physical activity in 4 studies (19, 30, 31, 33). Body weight, BMI, plasma glucose, TC were evaluated in all RCTs, insulin, TRGs, HDL-C, LDL-C were evaluated in 4 trials and SBP, DBP, WC, HOMA-IR were evaluated in 3 trials, body fat ratio was evaluated in 2 trials and fat mass, lean mass and HbA1c% were evaluated in one trial. The main characteristics of each included trial along with the outcomes studied are presented in Table 1.

**Table 1:** Study characteristics of the included randomized controlled trials.

Study	Year	Country	RCT design	Intervention (s)	Number of participants (Intervention)	Control (s)	Number of participants (Control)	Sample description	Age range	Follow up	Main outcomes
Akbulut et al. (30)	2021	Turkey	Parallel RCT	Med diet	30	Low-fat diet	30	Children newly diagnosed with NAFLD and with a BMI > 85th percentile	9 to 17 years	12 weeks	Weight, BMI, body fat ratio, TC, HDL-C, LDL-C, TRGs, glucose, insulin, HOMA-IR
Asoudeh et al. (18)	2023	Iran	Parallel RCT	Med diet	35	Standard diet	35	Adolescent girls with metabolic syndrome	13 to 18 years	12 weeks	Weight, BMI, WC, TC, HDL-C, LDL-C, TRGs, SBP, DBP, glucose, insulin, HOMA-IR
IGENOI study (32, 33)	2018	Spain	Parallel RCT	Med diet (moderately hypocaloric)	81	Standard diet	26	Children and adolescents with abdominal obesity	7 to 16 years	8 weeks	Weight, BMI, Glucose, insulin, SBP, DBP, TC
Velázquez-López et al. (31)	2014	Mexico	Parallel RCT	Med diet	24	Standard diet	25	Children and adolescents, with Body Mass Index (BMI) ≥ 95th percentile and any metabolic syndrome component	3 to 18 years	16 weeks	Weight, BMI, WC, fat mass, lean mass, SBP, DBP, TC, HDL-C, LDL-C, TRGs Glucose
Yurtdas et al. (19)	2021	Turkey	Parallel RCT	Med diet	22	Low-fat diet	22	Adolescents with BMI ≥ 95th percentile, diagnosed with grade ≥1 NAFLD	11 to 18 years	12 weeks	Weight, BMI, body fat ratio, WC, TC, HDL-C, LDL-C, TRGs, glucose, insulin, HbA1c%, HOMA-IR

BMI, body mass index; DBP, diastolic blood pressure; HDL-C, high density lipoprotein-concentration; HOMA-IR, homeostatic model assessment for insulin resistance; LDL-C, low density lipoprotein-concentration; Med diet, Mediterranean diet; NAFLD, Non-alcoholic fatty liver disease; RCTs, randomized controlled trial; SBP, systolic blood pressure; TC, total cholesterol; TRGs, triglycerides; WC, waist circumference.

### Anthropometric parameters

Body weight was assessed in 5 RCTs (18, 19, 30, 31, 33) with no statistically significant summary association (SMD = −0.21, 95% CI = −0.43 to 0.02, *I*^2^ = 0%). The results for BMI, assessed in 5 RCTs (18, 19, 30, 31, 33), showed a statistically significant summary association (SMD = −0.26, 95% CI = −0.4 to −0.03, *I*^2^ = 0%) with lower BMI in the Med diet group. WC was assessed in 3 RCTs (18, 19, 31) with non-significant summary association observed (SMD = −0.28, 95% CI = −0.68 to 0.11, *I*^2^ = 37.1%).

### Cardiometabolic parameters

SBP and DBP were assessed in 3 trials (18, 31, 33). The results for SBP showed a statistically significant summary association (SMD = −0.48, 95% CI = −0.87 to −0.09, *I*^2^ = 46%) with lower SBP in the Med diet group. On the other hand, non-significant summary association was observed for DBP (SMD = −0.10, 95% CI = -0.58 to 0.38, *I*^2^ = 65%).

TC was assessed in 5 RCTs (18, 19, 30, 31, 33) with non-significant summary association (SMD = −0.15, 95% CI = −0.42 to 0.12, *I*^2^ = 28.6%). Similar were the results for TRGs, HDL-C and LDL-C assessed in 4 trials (18, 19, 30, 31) (SMD = −0.17, 95% CI = −0.50 to 0.16, *I*^2^ = 35.9%; SMD = 0.41, 95% CI = −0.18 to 0.99, *I*^2^ = 78.4% and SMD = −0.12, 95% CI = −0.62 to 0.38, *I*^2^ = 71% respectively).

Glucose was assessed in 5 RCTs (18, 19, 30, 31, 33) with non-significant summary association (SMD = −0.29, 95% CI = −0.89 to 0.30, *I*^2^ = 84.7%). Insulin and HOMA-IR were assessed in 4 (18, 19, 30, 33) and 3 (18, 19, 30) trials, respectively, with non-significant summary associations (SMD = −0.07, 95% CI = −0.35 to 0.21, *I*^2^ = 18.3% and SMD = −0.31, 95% CI = −0.71 to 0.09, *I*^2^ = 42.9% respectively).

**Figure 2:**
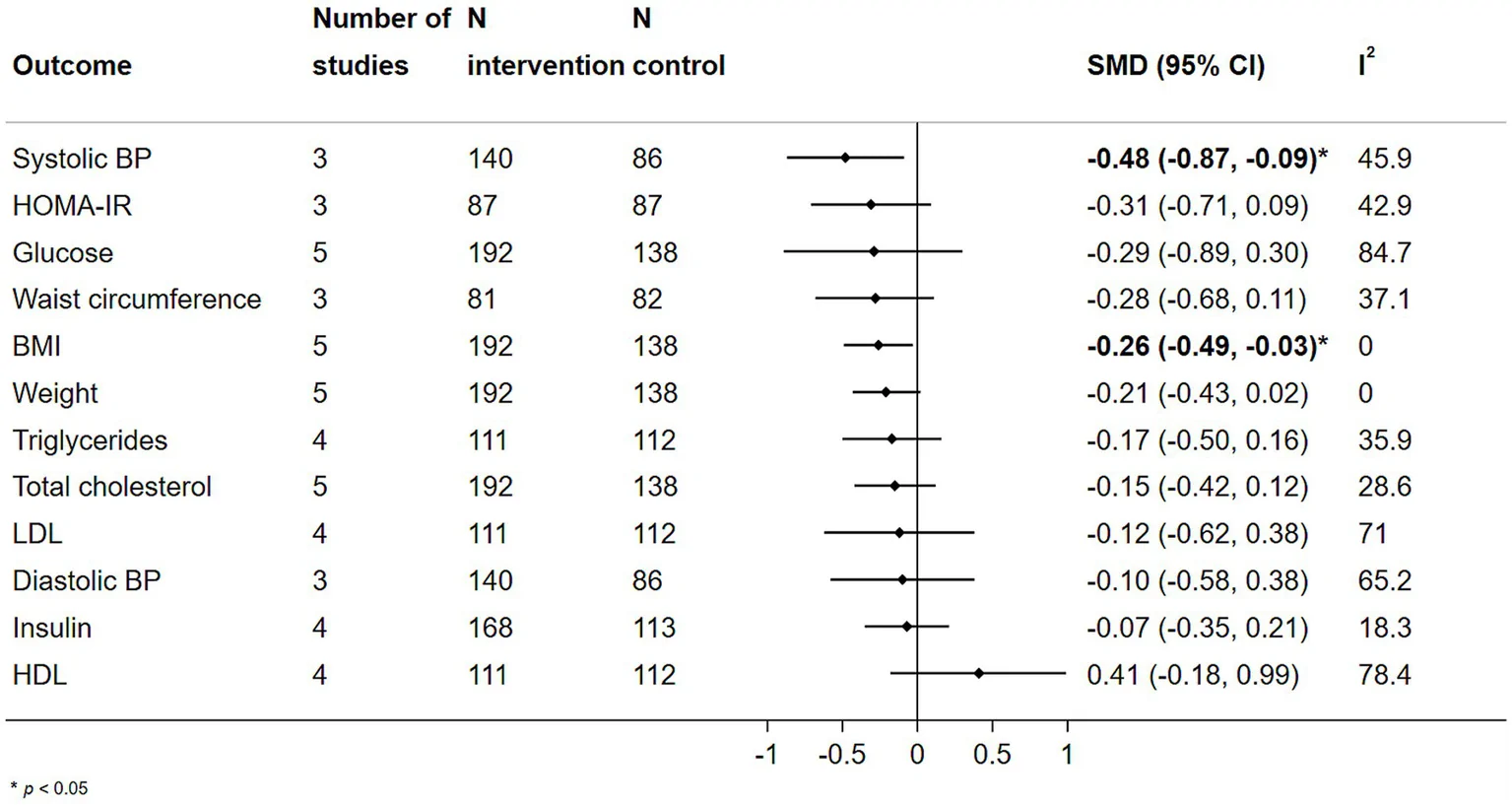
Forest plot of all meta-analyses of included randomized controlled trials by outcome.

All results are summarized in Figure 2 and Supplementary Table S5.

### Stratified analyses by type of control group

Overall, we observed no changes in the direction of the summary estimates with the following exceptions. For HOMA-IR, the results were significant for the comparison between Med diet and standard diet (1 RCT, SMD = −0.59, 95% CI = −1.07 to −0.11) and for HDL-C, the results were significant for the comparison between Med diet and standard diet (2 RCTs, SMD = 0.89, 95% CI = 0.51 to 1.26, *I*^2^ = 0%). This could be an indication of reduced efficacy of Med diet interventions once compared with an active control group (low-fat diet), but the number of studies is very limited to make any conclusions. The results of the post-hoc analysis stratified by type of control group are available on Supplementary Table S6.

### Sensitivity analyses

Overall, we observed some changes in the direction of the summary estimates when each study was individually removed from the analysis for the following comparisons. For SBP, the results were not significant when the study of Asoudeh F et al. (18) and the IGENOI study (33) were removed. These results could be related to participants’ and interventions’ characteristics as the study of Asoudeh F et al. (18) involved adolescents girls with abdominal obesity and metabolic syndrome, while the IGENOI study (33) involved children and adolescents with abdominal obesity who receive either a moderately hypocaloric Med diet or usual care. For BMI, the results were not significant when the study of Asoudeh F et al. (18) and the study of Yurtdas G et al. (19) were removed. These results could be related to participants’ characteristics as the study of Asoudeh F et al. (18) involved adolescents girls with abdominal obesity and metabolic syndrome, while the study of Yurtdas G et al. (19) involved children and adolescents with obesity (BMI ≥ 95th percentile) and non-alcoholic fatty liver disease. For WC, results were significant when the study of Velázquez-López L et al. (31) was removed. The study of Velázquez-López L et al. (31) involved children and adolescents with obesity (BMI ≥ 95th percentile) and metabolic syndrome. For HOMA-IR, results were significant when the study of Yurtdas G et al. (19) was removed. For HDL-C, results were significant when the study of Yurtdas G et al. (19) was removed. The study of Yurtdas G et al. (19) involved children and adolescents with obesity (BMI ≥ 95th percentile) and non-alcoholic fatty liver disease. The sensitivity analyses are available in detail in Supplementary Tables S7–S18.

### Risk of bias

All 5 RCTs (18, 19, 30, 31, 33) were judged to have some concerns based on the RoB 2.0 for each outcome assessed. All RCTs (18, 19, 30, 31, 33) were judged to raise some concerns with regard to the randomization process due to inadequate reporting of allocation sequence concealment and randomization process, 1 RCT (19) was judged to raise some concerns due to missing outcome data attributed to the high attrition rates during follow-up and 3 RCTs (18, 30, 31) were judged to raise some concerns in the selection of the reported results due to inadequate reporting of a detailed pre-specified plan of data analysis. The overall risk of bias and the risk of bias assessment for each domain is available on Table 2.

**Table 2:** Risk of bias summary for the included randomized controlled trials.

Study	Domain 1	Domain 2	Domain 3	Domain 4	Domain 5	Overall
Akbulut et al. (30)	Some concerns	Low risk	Low risk	Low risk	Some concerns	Some concerns
Asoudeh et al. (18)	Some concerns	Low risk	Low risk	Low risk	Some concerns	Some concerns
IGENOI study (32, 33)	Some concerns	Low risk	Low risk	Low risk	Low risk	Some concerns
Velázquez-López et al. (31)	Some concerns	Low risk	Low risk	Low risk	Some concerns	Some concerns
Yurtdas et al. (19)	Some concerns	Low risk	Some concerns	Low risk	Low risk	Some concerns

*Risk of bias assessment for each outcome attributed the same results.

Domain 1: Risk of bias arising from the randomization process.

Domain 2: Risk of bias due to deviations from the intended interventions.

Domain 3: Risk of bias due to missing outcome data.

Domain 4: Risk of bias in measurement of the outcome.

Domain 5: Risk of bias in selection of the reported result.

### Quality of evidence

Overall, the quality of evidence for summary estimates based on GRADE approach was judged as low quality for SBP, DBP, weight, BMI and insulin. The quality of evidence for summary estimates was judged as very low quality for WC, glucose, HOMA-IR, TC, TRGs, HDL-C, LDL-C. All results are summarized in Supplementary Table S19.

## Discussion

The aim of this systematic review and meta-analysis was to create a comprehensive overview of the randomized evidence examining the efficacy of the Med diet on various anthropometric and cardiometabolic parameters, focusing on children and adolescents affected by overweight and/or obesity, a population not separately represented in previous reports. Overall, this systematic review supports that Med diet could be associated with a beneficial effect on BMI and SBP reduction in children and adolescents with excess body weight. No significant associations were observed for any other anthropometric or cardiometabolic parameter. However, the results should be interpreted with caution because of the limited number of RCTs, the small sample sizes, the short follow-up period and the degree of heterogeneity in summary estimates for some outcomes, possibly explained by the variation between studies attributed to differences in interventions’ context, duration and intensity and patients’ characteristics.

### Comparison to previous evidence

Med diet is one of the most widely studied dietary patterns with supporting evidence of beneficial role in various anthropometric and cardiometabolic parameters related with non-communicable diseases such as diabetes mellitus and cardiovascular disease (5, 6, 39). This systematic review suggests that Med diet could be positively associated with greater reduction of BMI among children and adolescents with overweight or obesity, a finding supported by previous evidence synthesis. More specifically, previous systematic reviews and meta-analyses (40, 41), involving exclusively adult population, supported a greater BMI reduction among participants receiving Med diet-based interventions. Moreover, an umbrella review of systematic reviews reporting on 13 meta-analyses of observational studies and 16 meta-analyses of RCTs investigated the association between the adherence to Med diet and a number of different health outcomes showed a positive association between adherence to Med diet and BMI reduction (5). As far as children and adolescents are concerned, a recent meta-analysis of 15 RCTs, involving children and adolescents with normal and excess body weight, examined the efficacy of Med diet-based interventions on anthropometric parameters and obesity indicators (12). This meta-analysis (12) showed a statistically significant reduction of BMI among Med diet group participants, while according to a subgroup analysis, Med diet interventions were more effective among subjects with excess body weight both for BMI, WC, waist-to-height ratio, percentage of obesity, and percentage of abdominal obesity. In contrast to our meta-analysis that included studies focusing exclusively on dietary interventions based on Med diet, the abovementioned meta-analysis included not only studies with dietary interventions, but also studies with combined dietary and physical activity components that are more efficient in assessing the combined effect of Med diet and physical activity rather than the exclusive influence of Med diet as a distinct entity on the overall outcome observed. On the other hand, another systematic review that summarized evidence from 8 intervention studies and 47 observational (mainly cross-sectional) studies on the effectiveness of adherence to Med diet on adiposity markers and obesity among children and adolescents found limited evidence of a beneficial effect of adherence to Med diet and maintenance of a healthy body weight during childhood (42). Most of the studies included in this systematic review were of low quality. Again, the target population of this systematic review were children and adolescents, regardless of body weight (42). Also, the findings of an umbrella review of 4 systematic reviews and 3 meta-analyses including observational studies and RCTs focusing on the efficacy of Med diet adherence in various health outcomes among adolescents were inconclusive with regard to BMI, due to limited evidence and inconsistency (43). More specifically, the outcome of overweight and obesity was examined by 3 systematic reviews (42, 44, 45) with inconclusive results mainly attributed to observational study design that limited the assessment of cause-effect association, different definitions of obesity and obesity indicators across studies and lack of quantitative synthesis of the included studies (43).

There are several plausible mechanisms that could explain the beneficial role of Med diet in the reduction of BMI among children and adolescents with excess body weight. Firstly, some basic components of Med diet like fruits, vegetables, whole grain, nuts, poultry and fish are nutrient-dense foods with low calories that could contribute to increased satiety and lower energy intake (9, 46). In addition, Med diet, as a diet rich in antioxidants, vitamins, polyunsaturated fatty acids and polyphenols due to olive oil, fruit and vegetables consumption, has been associated with reduction in oxidative stress and systemic inflammation (47, 48), improved insulin resistance (49, 50) and modulated gut microbiota (51), mechanisms that are related to the pathogenesis of obesity (52, 53). Moreover, according to the new Med diet pyramid, Med diet encompasses preparing and sharing meals with family and friends (conviviality) (54–56). Conviviality involves complementary aspects beyond food like socialization during meals, self-awareness of satiety and hunger and meal satisfaction (56). Conviviality establishes a sense of community that could have a reinforcing role in the adherence to Med diet principles among children and adolescents. In addition, Med diet, as a holistic dietary pattern, encourages regular practice of moderate-to-intense physical activity (54, 55).

With regard to cardiometabolic parameters, previous evidence from systematic reviews and meta-analyses pertaining to adult population reported favorable results for Med diet efficacy on SBP (50, 57), DBP (50, 57), glucose (40, 41, 50), insulin resistance (40), HOMA-IR (50), TC (39–41), TRGs (41, 50) and HDL-C (41, 50). Considering children and adolescents, several observational studies showed mixed results (40, 58–64). Some reports showed favorable results in the incidence of hypertension (62, 63), hyperlipidemia (62, 63), obesity (58, 59, 63, 64) and metabolic syndrome (61–63) while another study found no association with metabolic syndrome (60). A recently published meta-analysis, including 9 RCTs that involved 577 children and adolescents regardless of body weight, examined the efficacy of Med diet on cardiometabolic parameters and reported a significant association between Med diet and SBP reduction, similarly to our findings (13). As long as elevated SBP is a risk factor for cardiovascular disease, a decrease of SBP during childhood and adolescence could also lead to a reduction of cardiovascular risk during adulthood (65). In addition, the abovementioned meta-analysis showed an association with reduction in TC, TRGs, LDL-C and an increase in HDL-C (13). Nevertheless, our meta-analysis found no association between Med diet and serum lipids levels, that could be related to smaller sample size and shorter mean studies’ duration. Longer duration of interventions would be plausible to reveal the possible beneficial effect on serum lipids and other biomarkers. Also, this meta-analysis (13), in accordance with our findings, found no association between Med diet and DBP, glucose, insulin or HOMA-IR. All results from the abovementioned meta-analysis pertain to children and adolescents, regardless of body weight, while no direct evidence on youths with excess body weight is reported.

### Strengths and limitations

To our knowledge, this systematic review is one of the first efforts to systematically collect, appraise and quantitively synthesize the available evidence from RCTs and create an inclusive map of randomized evidence on the efficacy of Med diet on a wide range of anthropometric and cardiometabolic parameters focusing exclusively on children and adolescents with overweight or obesity. Also, this systematic review included RCTs pertaining to Med diet as a holistic dietary pattern involving multiple food components and their possible interaction, as well as dietary counseling aiming at the adoption of a healthier lifestyle. This is because Med diet is a holistic lifestyle rather than the adoption of some of its components, and the possible association with health outcomes could exist for the overall diet and not necessarily for an isolated aspect of the Med diet (66).

Nevertheless, this systematic review entails certain limitations that should be considered. Firstly, only 5 RCTs involving 330 children and adolescents with excess body weight were included. The mean studies’ duration was 12 weeks ranging from 8 to 16 weeks. The short follow-up period could limit our ability to appraise the long-term effects of Med diet and the sustainability of the potential beneficial findings of BMI and SBP reduction. Short-term interventions (3–6 months duration) could have a beneficial role in cardiometabolic parameters like serum lipid profile and blood pressure, but long-term interventions are needed to estimate the potential beneficial effects on chronic conditions like obesity, diabetes mellitus and cardiovascular disease (39, 67, 68). Also, four RCTs (19, 30, 31, 33) incorporated physical activity recommendations for all study participants (both the intervention and the control group) that could influence positively the overall effect observed. In addition, data on the adherence to Med diet were limited as only two trials (19, 33) reported data on KIDMED, with higher scores in the Med diet group. Adherence to Med diet could impact the effectiveness of the intervention under study. Previous evidence supports that adherence to Med diet was related with improved lifestyle factors (physical activity, lower sedentary behavior, dietary adequacy) (44), lower risk of obesity (69) and metabolic syndrome (14). Another limitation that could reduce the validity of the results is the methodological quality of studies, as all RCTs were judged to have some methodological concerns mainly attributed to inadequate reporting of randomization process in all studies and the high attrition of participants in one trial (19). Regarding the synthesis of the available evidence, the small number of studies involving only 330 participants has limited the precision of the summary estimates as well as the ability to investigate any additional factors that could contribute to the variability of the results such as concomitant counseling on physical activity, differences between Mediterranean and non-Mediterranean countries, differences in participants’ characteristics (age, definition of obesity [BMI ≥ 95th percentile, abdominal obesity], presence of other health conditions [non-alcoholic fatty liver disease, metabolic syndrome]) or differences in the context (dietary counseling, provision of prescribed meals or combination, involvement of parents or guardians) or duration of the Med diet intervention. Moreover, the summary estimates may be interpreted within the context of high heterogeneity attributable to differences in intervention or control group characteristics (context, intensity or duration) and variance in population characteristics. Finally, summary estimates were calculated as SMDs, as pre-defined in the study protocol, to account for any possible differences in measurement scales used across studies. The use of SMDs, compared with mean differences, could be more challenging to interpret clinically.

### Implications for practice, policy and future research

Childhood obesity and its cardiometabolic complications pose a great health challenge worldwide. Our systematic review suggests a possible beneficial effect of Med diet on BMI and SBP among youths with overweight or obesity, indicating that Med diet could be an effective component of interventions targeting overweight and obesity during the early stages of life. Simultaneously, no associations with other anthropometric or cardiometabolic parameters were established. All results, both significant and non-significant findings, should be interpreted with caution because of the small sample size that may limit statistical power, the short studies’ duration, the variability in participants’ and interventions’ characteristics and the methodological limitations. Thus, this systematic review points out the need for high-quality RCTs, with higher number of participants, longer follow-up period, to better evaluate long-term effects of Med diet in younger population, and greater methodological rigor. Also, future studies may better address issues such as adherence to Med diet or include outcomes like quality of life that are poorly represented in the available literature. A recent systematic review and meta-analysis of RCTs investigating various types of interventions for children with central obesity showed that combining dietary and physical activity interventions could reduce central obesity, while dietary interventions alone were not effective (70). Obesity is a complex health issue requiring a combination of dietary, physical activity and behavioral interventions to be addressed. Toward that direction, further research is needed in the evaluation of Med diet in different contexts such as school-based or digital-based interventions or as part of a multidisciplinary intervention combining dietary, physical activity and behavioral methods interventions that would be part of a public health promotion program targeting obesity. In addition, further research, that may include real-world evidence, is highly requisite to elucidate the clinical relevance of the suggested beneficial effect of Med diet on BMI and SBP.

## Conclusion

This systematic review and meta-analysis of RCTs suggests that Med diet could be associated with lower BMI and SBP among children and adolescents affected by overweight or obesity. These results may be supportive of a potential beneficial effect of Med diet-based interventions in promoting a healthier lifestyle among youths with excess body weight. Nevertheless, taking into consideration the limited number of studies, the small sample size, the short studies’ duration and the methodological quality of trials, further research involving more participants, long-term interventions with extended post-intervention follow-up and greater methodological rigor is required to better understand the role of Med diet, alone or within the context of a multidisciplinary intervention, considering youths with overweight and obesity.

## Data Availability

The original contributions presented in the study are included in the article/Supplementary material, further inquiries can be directed to the corresponding authors.
