# Supplementary material for: Efficacy of Mediterranean diet interventions on children and adolescents affected by overweight or obesity: a systematic review and meta-analysis
Source: Front Nutr. 2026 Aug 26;13:1749319. doi: 10.3389/fnut.2026.1749319 (PMC13553484; doi:10.3389/fnut.2026.1749319)
Supplement: Supplementary file 1 [file Data_Sheet_1.DOCX]

**Table S1.** Search strategy.

| **Database** | **Search algorithm** |
| --- | --- |
| **PubMed** | ("Child"[Mesh] OR child* OR "Adolescent"[Mesh] OR adolescen*) AND ("Obesity"[Mesh] OR obes* OR "Overweight"[Mesh] OR overweight) AND ("Diet, Mediterranean"[Mesh] OR “Mediterranean diet” OR “Mediterranean dietary pattern”) AND (“randomised controlled trial” OR “randomized controlled trial” OR “randomised clinical trial” OR “randomized clinical trial” OR “clinical trial”)  **Results:**79 (Search until 15 September 2025) |
| **Cochrane Central Register of Controlled Trials (CENTRAL)** | "Mediterranean diet" AND (child* OR adolescen*) AND (obes* OR overweight)  **Results:**117 (Search until 10 September 2025) |
| **Scopus** | "Mediterranean diet" AND (children OR adolescent*) AND (overweight OR obes*) AND ("randomized controlled trial" OR "clinical trial")  **Results:**174 (Search until 07 June 2026) |

**Table S2.** Excluded studies with reasons for exclusion.

Mei S, Ding J, Wang K, Ni Z, Yu J. Mediterranean Diet Combined With a Low-Carbohydrate Dietary Pattern in the Treatment of Overweight Polycystic Ovary Syndrome Patients. Front Nutr. 2022 Apr 4;9:876620. doi: 10.3389/fnut.2022.876620. PMID: 35445067; PMCID: PMC9014200.

**Reason for exclusion:** Adult population

Santos-Beneit G, Fernández-Alvira JM, Tresserra-Rimbau A, Bodega P, de Cos-Gandoy A, de Miguel M, Ramírez-Garza SL, Laveriano-Santos EP, Arancibia-Riveros C, Carral V, Orrit X, Rodríguez C, Carvajal I, Haro D, Peyra C, Martínez-Gómez J, Álvarez-Benavides A, Estruch R, Lamuela-Raventós RM, Fernández-Jiménez R, Fuster V. School-Based Cardiovascular Health Promotion in Adolescents: A Cluster Randomized Clinical Trial. JAMA Cardiol. 2023 Sep 1;8(9):816-824. doi: 10.1001/jamacardio.2023.2231. PMID: 37531100; PMCID: PMC10398546.

**Reason for exclusion:** No overweight or obese children or adolescents

Prats-Arimon M, Puig-Llobet M, Barceló-Peiró O, Ribot-Domènech I, Vilalta-Sererols C, Fontecha-Valero B, Heras-Ojeda M, Agüera Z, Lluch-Canut T, Moreno-Poyato A, Moreno-Arroyo MC. An Interdisciplinary Intervention Based on Prescription of Physical Activity, Diet, and Positive Mental Health to Promote Healthy Lifestyle in Patients with Obesity: A Randomized Control Trial. Nutrients. 2024 Aug 20;16(16):2776. doi: 10.3390/nu16162776. PMID: 39203911; PMCID: PMC11357149.

**Reason for exclusion:** Adult population

Deshmukh A, Sood V, Lal BB, Khanna R, Alam S, Sarin SK. Effect of Indo-Mediterranean diet versus calorie-restricted diet in children with non-alcoholic fatty liver disease: A pilot randomized control trial. Pediatr Obes. 2024 Nov;19(11):e13163. doi: 10.1111/ijpo.13163. Epub 2024 Sep 2. PMID: 39223952.

**Reason for exclusion:** Control group similar to Mediterranean diet

Martíncrespo-Blanco MC, Varillas-Delgado D, Blanco-Abril S, Cid-Exposito MG, Robledo-Martín J. Effectiveness of an Intervention Programme on Adherence to the Mediterranean Diet in a Preschool Child: A Randomised Controlled Trial. Nutrients. 2022 Apr 7;14(8):1536. doi: 10.3390/nu14081536. PMID: 35458098; PMCID: PMC9025428.

**Reason for exclusion:** No Mediterranean diet intervention

Morales E, Torres-Castillo N, Garaulet M. Infancy and Childhood Obesity Grade Predicts Weight Loss in Adulthood: The ONTIME Study. Nutrients. 2021 Jun 22;13(7):2132. doi: 10.3390/nu13072132. PMID: 34206431; PMCID: PMC8308354.

**Reason for exclusion:** No RCT

Catalán-Lambán A, Ojeda-Rodríguez A, Marti Del Moral A, Azcona-Sanjulian C. Changes in objectively measured sleep after a multidisciplinary lifestyle intervention in children with abdominal obesity: A randomized trial. Sleep Med. 2023 Sep;109:252-260. doi: 10.1016/j.sleep.2023.07.004. Epub 2023 Jul 17. PMID: 37487278.

**Reason for exclusion:** No outcome of interest

Bibiloni MDM, Fernández-Blanco J, Pujol-Plana N, Surià Sonet S, Pujol-Puyané MC, Mercadé Fuentes S, Ojer Fernández de Soto L, Tur JA. Reversión de sobrepeso y obesidad en población infantil de Vilafranca del Penedès: Programa ACTIVA’T (2012) [Reversion of overweight and obesity in Vilafranca del Penedès child population: ACTIVA'T Program (2012)]. Gac Sanit. 2019 Mar-Apr;33(2):197-202. Spanish. doi: 10.1016/j.gaceta.2017.10.002. Epub 2017 Nov 21. PMID: 29169902.

**Reason for exclusion:** No RCT

Arenaza L, Medrano M, Oses M, Amasene M, Díez I, Rodríguez-Vigil B, Labayen I. The Effect of a Family-Based Lifestyle Education Program on Dietary Habits, Hepatic Fat and Adiposity Markers in 8-12-Year-Old Children with Overweight/Obesity. Nutrients. 2020 May 16;12(5):1443. doi: 10.3390/nu12051443. PMID: 32429379; PMCID: PMC7284532.

**Reason for exclusion:** No Mediterranean diet intervention

Macknin M, Stegmeier N, Thomas A, Worley S, Li L, Hazen SL, Tang WHW. Three Healthy Eating Patterns and Cardiovascular Disease Risk Markers in 9 to 18 Year Olds With Body Mass Index >95%: A Randomized Trial. Clin Pediatr (Phila). 2021 Oct;60(11-12):474-484. doi: 10.1177/00099228211044841. Epub 2021 Sep 21. PMID: 34546139; PMCID: PMC8919177.

**Reason for exclusion:** Control group similar to Mediterranean diet

Morell-Azanza L, Ojeda-Rodríguez A, Azcona-SanJulián MC, Zalba G, Marti A. Associations of telomere length with anthropometric and glucose changes after a lifestyle intervention in abdominal obese children. Nutr Metab Cardiovasc Dis. 2020 Apr 12;30(4):694-700. doi: 10.1016/j.numecd.2019.12.002. Epub 2019 Dec 10. PMID: 32007334.

**Reason for exclusion:** No RCT

La Torre G, Mannocci A, Saulle R, Sinopoli A, D'Egidio V, Sestili C, Manfuso R, Masala D. [GiochiAMO! The protocol of a school based intervention for the promotion of physical activity and nutrition among children]. Clin Ter. 2016 Sep-Oct;167(5):152-155. Italian. doi: 10.7417/CT.2016.1947. PMID: 27845482.

**Reason for exclusion**: Study protocol

Fernández-Ruiz VE, Solé-Agustí M, Armero-Barranco D, Cauli O. Weight Loss and Improvement of Metabolic Alterations in Overweight and Obese Children Through the I^2^AO^2^ Family Program: A Randomized Controlled Clinical Trial. Biol Res Nurs. 2021 Jul;23(3):488-503. doi: 10.1177/1099800420987303. Epub 2021 Feb 1. PMID: 33517762.

**Reason for exclusion:** No Mediterranean diet intervention

Sofi F, Dinu M, Pagliai G, Cesari F, Marcucci R, Casini A. Mediterranean versus vegetarian diet for cardiovascular disease prevention (the CARDIVEG study): study protocol for a randomized controlled trial. Trials. 2016 May 4;17(1):233. doi: 10.1186/s13063-016-1353-x. Erratum in: Trials. 2016 May 19;17(1):253. doi: 10.1186/s13063-016-1395-0. PMID: 27145958; PMCID: PMC4855805.

**Reason for exclusion**: Study protocol

**Abbreviations:** RCT:randomized controlled trial

**Table S3.** Detailed description of the participants’ inclusion and exclusion criteria of the included randomized controlled trials.

| **Study** | **Inclusion/Exclusion criteria of study participants** |
| --- | --- |
| Akbulut UE. et al.[18] | **Inclusion criteria:** Age 9–17 years, body mass index (BMI) z-score >85th percentile, diagnosis of NAFLD [alanine aminotransferase (ALT) levels greater than twice the upper limit of the normal level (males > 50 U/L, females > 44 U/L) and fatty liver disease detected at ultrasonography]. |
|  | **Exclusion criteria:** Presence of diabetes, secondary causes of NAFLD (e.g. medication induced), current use of weight loss medications, and other causes of fatty liver disease (such as Wilson disease, alpha-1 antitrypsin deficiency, autoimmune hepatitis, and viral hepatitis). Patients with history of alcohol consumption were not included in the study, and the patients were not allowed to consume alcohol during it. In addition, children who had previously presented to paediatric or paediatric obesity clinics and who had received any dietary treatment were excluded from the study. |
| Asoudeh F. et al.[16] | **Inclusion criteria:** Adolescent girls, with metabolic syndrome (as defined below), aged 13–18 years, who was not on a specific diet in the last 3 months. Metabolic syndrome was defined based on International Diabetes Federation (IDF) criteria as: abdominal obesity (waist circumference above the 90th percentile) plus having two or more of the following characteristics: (1) Elevated blood pressure (systolic pressure equal or greater than 130 mmHg diastolic blood pressure equal to or above 85 mmHg, (2) high-serum triglyceride levels (equal to or greater than 150 mg/dL), (3) abnormal fasting plasma glucose (equal to or greater than 100 mg/dL), and (4) low-serum HDL cholesterol levels (below 40 mg/dL). |
|  | **Exclusion criteria:** Individuals who were on a specific diet or those who were taking medications that affect appetite and weight, individuals who were taking multivitamin or mineral supplements, omega-3 fat supplements, and were using drugs affecting carbohydrate metabolism, fat and blood pressure |
| IGENOI study [15,19] | **Inclusion criteria:** Children aged 7 to 16 years, with a waist circumference above the 90th percentile according to national data. |
|  | **Exclusion criteria:** Prevalent pre-diabetes, food intolerance, eating disorders or psychiatric disease, pharmacological treatment, regular alcohol consumption or special diet treatment |
| Velázquez-López L. et al.[20] | **Inclusion criteria:** Body Mass Index (BMI) ≥95th percentile and any metabolic syndrome component, according to modified International Diabetes Federation (IDF) criteria for children and adolescents; waist circumference (WC) ≥90th percentile; fasting glucose ≥100 mg/dL; TG ≥150 mg/dL; HDL-C ≤ 40 mg/dL; systolic blood pressure (SBP) ≥130 mmHg and/ or diastolic blood pressure (DBP) ≥85 mmHg. |
|  | **Exclusion criteria:** Presence of chronic illness, use of pharmacological treatment for obesity and/or its comorbidities that would limit the participation in the dietary intervention. |
| Yurtdas G. et al.[17] | **Inclusion criteria:** Age between 11 and 18 years old, body mass index (BMI) ≥95th percentile, and being diagnosed with grade ≥1 NAFLD. |
|  | **Exclusion criteria:** Presence of other hepatic diseases (such as hepatitis), alcohol consumption, history of type I or type II diabetes, use of drugs that may affect the liver fat or lipid profile, dietary treatment for weight loss, presence of chronic inflammatory disease, cancer, thyroid dysfunction (hyperthyroidism and hypothyroidism), pregnancy or lactation, antibiotic treatment within 3 months prior to the study, and use nutritional supplements. Patients positive for chronic hepatitis other than NAFLD according to past medical data were excluded from the study. |

**Abbreviations:** NAFLD: Non-alcoholic fatty liver disease

**Table S4.** Detailed description of the intervention and the control group of the included randomized controlled trials.

| **Study** | **Description of Intervention/Control group** |
| --- | --- |
| Akbulut UE. et al.[18] | **Intervention:** At the baseline clinic visit, all children underwent a 30-min individual education session. Weekly meal plans were produced collaboratively with the assistance of a registered dietician and were tailored to family preferences. During the education session, children received nutritional recommendations and a food-group list specifying preferred choices and approximate numbers and size of servings per day based on dietary modelling. They were also told which foods should be avoided and were given potential alternatives with variable equicaloric values. The patients were instructed not to consume anything other than the recommended foods. Energy expenditure was calculated using the Schofield equation adapted for age and gender. The percentage of calorie restriction varied from 10 to 20% of total energy intake, values which did not interfere with the children’s body growth.  The MeD is rich in plant-based foods including vegetables, whole cereals, and fruit, the main added fat is extra virgin olive oil. The diet involves high levels of consumption of legumes, nuts, and fish, low consumption of red meat and home-made desserts, and moderate consumption of fermented milk  and poultry. The MeD had a target macronutrient composition of 35–40% fat  (with 10% of energy constituting saturated fat), 40–44% carbohydrate, and 20% protein. |
|  | **Control:** At the baseline clinic visit, all children underwent a 30-min individual education session. Weekly meal plans were produced collaboratively with the assistance of a registered dietician and were tailored to family preferences. During the education session, children received nutritional recommendations and a food-group list specifying preferred choices and approximate numbers and size of servings per day based on dietary modelling. They were also told which foods should be avoided and were given potential alternatives with variable equicaloric values. The patients were instructed not to consume anything other than the recommended foods. Energy expenditure was calculated using the Schofield equation adapted for age and gender. The percentage of calorie restriction varied from 10 to 20% of total energy intake, values which did not interfere with the children’s body growth.  The low-fat diet had a target macronutrient composition of 55% of energy from carbohydrates, 20–25% from fat (with 10% of energy consisting of saturated fat), and 20–25% from protein. Nutrition education focused on the selection of foods containing 3g of fat/serving, limiting added fats, and using low-fat meal preparation strategies. Parents were instructed to provide ample quantities of grains, vegetables, fruits, lean meats, and low-fat dairy products and to restrict high-fat foods. |
|  | **Physical activity recommendation:** In the beginning, each child underwent a motivational interview at which the disadvantages of inactivity and the benefits of mobility in terms of eliminating potential exercise intolerance and reluctance were explained. In both intervention and control group, children participated in a 12-week exercise, program for 30–45 min/3 days in the first two weeks and for 60 min/4–5 days in the following weeks. They were asked to select “one or more” moderate-intensity aerobic exercises (brisk walking, jogging, football, basketball, cycling, swimming, gymnastics, and dancing), which they found enjoyable. They were also encouraged to significantly reduce sedentary behaviors (prolonged sitting and extended screen-time, such as watching television, computers, phones, tablets, and game consoles) and to  be more physically active in daily life and school (being active during school breaks, playing in the park, participating in street games, walking with pets, using stairs instead of lifts, etc.). The motivational interview was repeated at the children’s monthly follow-up visits. |
| Asoudeh F. et al.[16] | **Intervention:** MeD was designed firstly by computing energy requirements for each study participant based on his/her weight by adding 2 kcal per gram of weight gain. Then, macronutrient composition was determined, in which they planned each menu to have 53– 58% of energy from carbohydrates, 15–18% from proteins, and 26–30% from dietary fats. In each dietary menu, increased consumption of olive oil, fruit, vegetables, and low-fat dairy products was recommended while reducing total saturated fat intake and cholesterol intake was requested. Participants were also requested to replace refined grains with dietary sources of whole grains. In addition, low consumption of sweets and red meat was recommended in these dietary menus. Along with these menus, each participant was given an exchange list in which the exchanges for food items included in the menus were given. Participants were instructed how to use these exchange lists. |
|  | **Control:** Participants were only advised verbally to follow healthy eating tips  recommended according to the usual Iranian diet. |
|  | **Physical activity recommendation:** None reported. |
| IGENOI study[15,19] | **Intervention:** Intensive care participants followed a Mediterranean-style diet  based on high consumption of fruit, vegetables, whole grains, legumes, nuts, seeds and olive oil, minimally processed foods; moderate consumption of dairy products, fish and poultry; and low consumption of red meat. The intensive care group was advised to follow a moderately hypocaloric MeD. On the first visit, intensive care participants were prescribed to follow a diet based on a fixed full-day meal plan. The energy expenditure was calculated using the Schofield equation adapted to age and sex. The percentage of calorie restriction varied from 10% to 40% of total energy intake depending on the degree of obesity presented, which did not interfere with the children’s body growth. In all cases, the caloric ranges of diets were between 1300 and 2200 kcal. The diet was distributed into five main meals: breakfast providing 20%, morning snack 5–10%, lunch 30– 35%, afternoon snack 10–15% and dinner 20–25% of total energy. The distribution of carbohydrate, fat and proteins was 55%, 30% and 15% of total energy, respectively. During the eight weeks period, participants assigned to the intensive care group completed six 30-min individual sessions with the research team. Additionally, participants had a group meeting. Dieticians provided information on healthy lifestyle and how to manage obesity-related problems to parents and legal guardians. Children were taught several topics such as controlling healthy lifestyle behavior, food preparation, portion control, eating behavior, food composition and the importance of being physically active (by increasing physical activity and reducing sedentary time) during leisure time. |
|  | **Control:** The usual group received standard paediatric recommendations on healthy diet. During the eight-week period, usual care subjects received a 30- min individual session with the dietician and five monitoring visits to assess anthropometric parameters. |
|  | **Physical activity recommendation:** Both intervention and control group were encouraged to accumulate an extra time of 200 min of physical activity per week at 60–75% of their maximum heart rate. |
| Velázquez-López L. et al.[20] | **Intervention:** MeD included the following recommendations: a) 60% of carbohydrates (50% complex and no more than 10% refined and processed sugars), 25% lipids, and 15% proteins. It was also recommended that they consume foods rich in a) essential fatty acids, such as safflower, corn, olive, and soy oils; b) omega 3 fatty acids (alphalinolenic acid, eicosapentaenoic acid, and docosahexaenoic acid, found in foods such as salmon, mackerel, tuna, grouper, anchovies, flaxseed, canola, walnuts, and wheat germ.; c) omega 9 fatty acids (oleic and erucic acids), found in olive and canola oils, as well as Erysimum and mustard seeds; d) antioxidants, such as beta carotenes, lycopenes, vitamin A, vitamin C (found in papaya, strawberries, kiwi, oranges and other citrus fruits, green and red peppers, broccoli, spinach, and raw tomatoes), vitamin E, selenium, zinc, copper, and flavonoids (found in grapes, apples, cherries, broccoli, radishes, beets, seeds, flowers, green tea, black tea, soy, Ginkgo biloba, thistle, and cranberries); e) fiber (found in fruits and cereals). Also, participants received specific menus including the aforementioned food items. In accordance with the calories corresponding to them, each patient was encouraged to consume fresh fruit and vegetables, while any dairy products and products of animal origin should be low in fat. It was recommended that they limit their consumption of fried food, while the consumption of steamed foods was encouraged. It was suggested that eating times be respected and that food should preferably be consumed at home. It was also suggested that the consumption of fast food be limited to once a month or less. The consumption of sugary drinks and packaged juices was limited, while the consumption of natural water was encouraged along with drinks prepared using fruit but no added sugar. The diet was administered using a food equivalents system. Food replicas were used so that patients and their parents/guardians could learn to recognize a healthy diet, and menus were tailored according to the age- and gender-required calories of each participant. Participants received their diet plans in writing, containing a graphic representation of the food groups, menu proto-types and general recommendations for healthy nutrition. To determine the participants’ typical diet prior to the intervention, a 24-hour recalls was used. Participants were evaluated every three weeks over the intervention to measure diet compliance and reinforce the MeD. |
|  | **Control:** Standard diet consisted of 55-60% of carbohydrates (45-50% complex and no more than 10% refined and processed sugars), 25-30% lipids, and 15% proteins). In accordance with the calories corresponding to them, each patient was encouraged to consume fresh fruit and vegetables, while any dairy products and products of animal origin should be low in fat. It was recommended that they limit their consumption of fried food, while the consumption of steamed foods was encouraged. It was suggested that eating times be respected and that food should preferably be consumed at home. It was also suggested that the consumption of fast food be limited to once a month or less. The consumption of sugary drinks and packaged juices was limited, while the consumption of natural water was encouraged along with drinks prepared using fruit but no added sugar. The diet was administered using a food equivalents system. Food replicas were used so that patients and their parents/guardians could learn to recognize a healthy diet, and menus were tailored according to the age- and gender-required calories of each participant. Participants received their diet plans in writing, containing a graphic representation of the food groups, menu proto-types and general recommendations for healthy nutrition. Participants were evaluated every three weeks to measure diet compliance and reinforce the standard diet. |
|  | **Physical activity recommendation:** Both intervention and control groups received general recommendations about performing physical activity. |
| Yurtdas G. et al.[17] | **Intervention:** Target macronutrient energy contributions were 40% from carbohydrates, 35%–40% from fat (with <10% from saturated fat), and 20% from protein. Participants in this group were instructed to consume fish and, legumes at least 2–3 times a week, and walnuts (20 g/day), and olive oil (30–45 g/day) every day, in accordance with the MeD model. MeD was designed to be non-calorie restricted. In order to ensure that both study groups were able to apply the recommended diets correctly and appropriately, the dietitian had a phone call with the participants and their parents twice a week and continuous communication was established via social media. Daily meal plans were created in accordance with the energy intake of adolescents and sent to participants over social media for 12 weeks. Adolescents were invited to the research center to be checked every 2 weeks, and 3-day dietary records were kept by all of the adolescents with the help of their parents. |
|  | **Control:** Target macronutrient energy contributions for the low-fat diet were 50%–60% from carbohydrates, <30% from fat (with <10% from saturated fat), and 20% from protein. Participants in this group were especially recommended to consume low-fat foods. Dietary principles to be followed in nonalcoholic fatty liver disease such as restricting saturated fat intake, avoiding processed/packaged products, alcohol, instant beverages, carbonated/sugary drinks, and sweets containing high amounts of sugar and fat, were explained to adolescents in both diet groups by the same research dietitian. Low-fat diet was designed to be non-calorie restricted. In order to ensure that both study groups were able to apply the recommended diets correctly and appropriately, the dietitian had a phone call with the participants and their parents twice a week and continuous communication was established via social media. Daily meal plans were created in accordance with the energy intake of adolescents and sent to participants over social media for 12 weeks. Adolescents were invited to the research center to be checked every 2 weeks, and 3-day dietary records were kept by all of the adolescents with the help of their parents. |
|  | **Physical activity recommendation:** Participants were advised to maintain their usual level of activity for the duration of the intervention. |

**Abbreviations:** MeD: Mediterranean diet

**Table S5.** Summary results of the included RCTs.

| **Outcome** | **N studies** | **N intervention** | **N control** | **SMD** | **95% Confidence Intervals** | **I^2^ (%)** | **Egger’s p-value** | **Meta-regression p-value for exercise prescription** |
| --- | --- | --- | --- | --- | --- | --- | --- | --- |
| Systolic BP | 3 | 140 | 86 | -0.48 | -0.87 to -0.09 | 45.9 | NA | NA |
| Diastolic BP | 3 | 140 | 86 | -0.10 | -0.58 to 0.38 | 65.2 | NA | NA |
| Weight | 5 | 192 | 138 | -0.21 | -0.43 to 0.02 | 0 | NA | NA |
| BMI | 5 | 192 | 138 | -0.26 | -0.49 to -0.03 | 0 | NA | NA |
| WC | 3 | 81 | 82 | -0.28 | -0.68 to 0.11 | 37.1 | NA | NA |
| Glucose | 5 | 192 | 138 | -0.29 | -0.89 to 0.30 | 84.7 | NA | NA |
| Insulin | 4 | 168 | 113 | -0.07 | -0.35 to 0.21 | 18.3 | NA | NA |
| HOMA-IR | 3 | 87 | 87 | -0.31 | -0.71 to 0.09 | 42.9 | NA | NA |
| TC | 5 | 192 | 138 | -0.15 | -0.42 to 0.12 | 28.6 | NA | NA |
| TRGs | 4 | 111 | 112 | -0.17 | -0.50 to 0.16 | 35.9 | NA | NA |
| LDL-C | 4 | 111 | 112 | -0.12 | -0.62 to 0.38 | 71 | NA | NA |
| HDL-C | 4 | 111 | 112 | 0.41 | -0.18 to 0.99 | 78.4 | NA | NA |

**Abbreviations:** BMI: body mass index, BP:blood pressure, HDL-C:high density lipoprotein-concentration, HOMA-IR: homeostatic model assessment for insulin resistance, LDL-C:low density lipoprotein-concentration, N: number, NA: not applicable, RCTs: randomized controlled trials, SMD:standardized mean difference,TC: total cholesterol, TRGs:triglycerides, WC: waist circumference

**Figure S1.** Comparison of Mediterranean diet versus control diet for systolic blood pressure.

**Figure S2.** Comparison of Mediterranean diet versus control diet for diastolic blood pressure.

**Figure S3.** Comparison of Mediterranean diet versus control diet for weight.

**Figure S4.** Comparison of Mediterranean diet versus control diet for body mass index (BMI).

**Figure S5.** Comparison of Mediterranean diet versus control diet for waist circumference.

**Figure S6.** Comparison of Mediterranean diet versus control diet for glucose.

**Figure S7.** Comparison of Mediterranean diet versus control diet for insulin.

**Figure S8.** Comparison of Mediterranean diet versus control diet for homeostatic model assessment for insulin resistance (HOMA-IR).

**Figure S9.** Comparison of Mediterranean diet versus control diet for total cholesterol.

**Figure S10.** Comparison of Mediterranean diet versus control diet for triglycerides.

**Figure S11.** Comparison of Mediterranean diet versus control diet for low density cholesterol (LDL-C).

**Figure S12.** Comparison of Mediterranean diet versus control diet for high density cholesterol (HDL-C).

**Table S6.** Summary results of the included randomized controlled trials stratified by control group.

|  | **Low-fat diet** | | | | | | **Standard diet** | | | | | |
| --- | --- | --- | --- | --- | --- | --- | --- | --- | --- | --- | --- | --- |
| **Outcome** | **N studies** | **N intervention** | **N control** | **SMD** | **95% Confidence Intervals** | **I^2^(%)** | **N studies** | **N intervention** | **N control** | **SMD** | **95% Confidence Intervals** | **I^2^(%)** |
| Systolic BP | NA | NA | NA | NA | NA | NA | 3 | 140 | 86 | -0.48 | -0.87 to  -0.09 | 45.9 |
| Diastolic BP | NA | NA | NA | NA | NA | NA | 3 | 140 | 86 | -0.10 | -0.58 to 0.38 | 65.2 |
| Weight | 2 | 52 | 52 | -0.27 | -0.65 to 0.12 | 0 | 3 | 140 | 86 | -0.17 | -0.45 to 0.11 | 0 |
| BMI | 2 | 52 | 52 | -0.32 | -0.71 to 0.06 | 0 | 3 | 140 | 86 | -0.22 | -0.50 to 0.06 | 0 |
| WC | 2 | 52 | 52 | -0.16 | -0.66 to 0.34 | 46.8 | 1 | 22 | 22 | -0.59 | -1.19 to 0.02 | NA |
| Glucose | 2 | 52 | 52 | -0.01 | -0.39 to 0.38 | 0 | 3 | 140 | 86 | -0.50 | -1.51 to 0.52 | 91.6 |
| Insulin | 2 | 52 | 52 | -0.01 | -0.39 to 0.38 | 0 | 2 | 116 | 61 | -0.13 | -0.71 to 0.45 | 68.9 |
| HOMA-IR | 2 | 52 | 52 | -0.15 | -0.64 to 0.35 | 37.8 | 1 | 35 | 35 | -0.59 | -1.07 to  -0.11 | NA |
| TC | 2 | 52 | 52 | -0.12 | -0.50 to 0.27 | 0 | 3 | 140 | 86 | -0.19 | -0.66 to 0.27 | 62.7 |
| TRGs | 2 | 52 | 52 | -0.03 | -0.41 to 0.36 | 0 | 2 | 59 | 60 | -0.33 | -1.04 to 0.37 | 72.2 |
| LDL-C | 2 | 52 | 52 | 0.12 | -0.44 to 0.68 | 51.3 | 2 | 59 | 60 | -0.35 | -1.27 to 0.57 | 83.5 |
| HDL-C | 2 | 52 | 52 | -0.06 | -0.71 to 0.59 | 63.7 | 2 | 59 | 60 | 0.89 | 0.51 to 1.26 | 0 |

**Abbreviations:** BMI: body mass index, BP:blood pressure, HDL-C:high density lipoprotein-concentration, HOMA-IR: homeostatic model assessment for insulin resistance, LDL-C:low density lipoprotein-concentration, N: number, NA: not applicable, SMD:standardized mean difference,TC: total cholesterol, TRGs:triglycerides, WC: waist circumference

**Table S7.** Sensitivity analyses for systolic blood pressure excluding one by one each randomized controlled trial.

| **Study** | **SMD** | **95% Confidence Intervals** | **I^2^ (%)** |
| --- | --- | --- | --- |
| Reference | -0.48 | -0.87 to -0.09 | 45.9 |
| Omitting Asoudeh F | -0.03 | -0.59 to 0.53 | 62.7 |
| Omitting Velázquez-López L | -0.67 | -0.99 to -0.34 | 0 |
| Omitting IGENOI study | -0.38 | -1.04 to 0.27 | 68.5 |

**Abbreviations:** SMD:standardized mean difference

**Table S8.** Sensitivity analyses for diastolic blood pressure excluding one by one each randomized controlled trial.

| **Study** | **SMD** | **95% Confidence Intervals** | **I^2^ (%)** |
| --- | --- | --- | --- |
| Reference | -0.10 | -0.58 to 0.38 | 65.2 |
| Omitting Asoudeh F | -0.34 | -0.70 to 0.02 | 5.4 |
| Omitting Velázquez-López L | -0.09 | -0.87 to 0.68 | 82.6 |
| Omitting IGENOI study | 0.13 | -0.28 to 0.53 | 20.2 |

**Abbreviations:** SMD:standardized mean difference

**Table S9.** Sensitivity analyses for weight excluding one by one each randomized controlled trial.

| **Study** | **SMD** | **95% Confidence Intervals** | **I^2^ (%)** |
| --- | --- | --- | --- |
| Reference | -0.21 | -0.43 to 0.02 | 0 |
| Omitting Akbulut UE | -0.22 | -0.47 to 0.04 | 0 |
| Omitting Asoudeh F | -0.19 | -0.63 to 0.26 | 0 |
| Omitting Velázquez-López L | -0.24 | -0.49 to 0.01 | 0 |
| Omitting IGENOI study | -0.21 | -0.48 to 0.05 | 0 |
| Omitting Yurtdas G | -0.17 | -0.42 to 0.07 | 0 |

**Abbreviations:** SMD:standardized mean difference

**Table S10.** Sensitivity analyses for body mass index (BMI) excluding one by one each randomized controlled trial.

| **Study** | **SMD** | **95% Confidence Intervals** | **I^2^ (%)** |
| --- | --- | --- | --- |
| Reference | -0.26 | -0.49 to -0.03 | 0 |
| Omitting Akbulut UE | -0.27 | -0.53 to -0.02 | 0 |
| Omitting Asoudeh F | -0.21 | -0.47 to 0.05 | 0 |
| Omitting Velázquez-López L | -0.28 | -0.53 to -0.03 | 0 |
| Omitting IGENOI study | -0.31 | -0.58 to -0.05 | 0 |
| Omitting Yurtdas G | -0.22 | -0.46 to 0.03 | 0 |

**Abbreviations:** SMD:standardized mean difference

**Table S11.** Sensitivity analyses for waist circumference excluding one by one each randomized controlled trial.

| **Study** | **SMD** | **95% Confidence Intervals** | **I^2^ (%)** |
| --- | --- | --- | --- |
| Reference | -0.28 | -0.68 to 0.11 | 37.1 |
| Omitting Asoudeh F | -0.22 | -0.92 to 0.47 | 64.9 |
| Omitting Velázquez-López L | -0.46 | -0.84 to -0.09 | 0 |
| Omitting Yurtdas G | -0.16 | -0.66 to 0.34 | 46.8 |

**Abbreviations:** SMD:standardized mean difference

**Table S12.** Sensitivity analyses for glucose excluding one by one each randomized controlled trial.

| **Study** | **SMD** | **95% Confidence Intervals** | **I^2^ (%)** |
| --- | --- | --- | --- |
| Reference | -0.29 | -0.89 to 0.30 | 84.7 |
| Omitting Akbulut UE | -0.35 | -1.12 to 0.42 | 88.3 |
| Omitting Asoudeh F | -0.13 | -0.76 to 0.50 | 82.2 |
| Omitting Velázquez-López L | -0.11 | -0.71 to 0.50 | 75.8 |
| Omitting IGENOI study | -0.50 | -1.06 to 0.07 | 76.6 |
| Omitting Yurtdas G | -0.39 | -1.12 to 0.34 | 74.8 |

**Abbreviations:** SMD:standardized mean difference

**Table S13.** Sensitivity analyses for insulin excluding one by one each randomized controlled trial.

| **Study** | **SMD** | **95% Confidence Intervals** | **I^2^ (%)** |
| --- | --- | --- | --- |
| Reference | -0.07 | -0.35 to 0.21 | 18.3 |
| Omitting Akbulut UE | -0.06 | -0.45 to 0.33 | 49.5 |
| Omitting Asoudeh F | 0.06 | -0.23 to 0.36 | 0 |
| Omitting IGENOI study | -0.17 | -0.48 to 0.13 | 6 |
| Omitting Yurtdas G | -0.11 | -0.46 to 0.23 | 38 |

**Abbreviations:** SMD:standardized mean difference

**Table S14.** Sensitivity analyses for homeostatic model assessment for insulin resistance (HOMA-IR) excluding one by one each randomized controlled trial.

| **Study** | **SMD** | **95% Confidence Intervals** | **I^2^ (%)** |
| --- | --- | --- | --- |
| Reference | -0.31 | -0.71 to 0.09 | 42.9 |
| Omitting Akbulut UE | -0.25 | -0.96 to 0.45 | 71.1 |
| Omitting Asoudeh F | -0.15 | -0.64 to 0.35 | 37.8 |
| Omitting Yurtdas G | -0.49 | -0.84 to -0.14 | 0 |

**Abbreviations:** SMD:standardized mean difference

**Table S15.** Sensitivity analyses for total cholesterol excluding one by one each randomized controlled trial.

| **Study** | **SMD** | **95% Confidence Intervals** | **I^2^ (%)** |
| --- | --- | --- | --- |
| Reference | -0.15 | -0.42 to 0.12 | 28.6 |
| Omitting Akbulut UE | -0.19 | -0.54 to 0.15 | 44.6 |
| Omitting Asoudeh F | -0.22 | -0.54 to 0.09 | 32 |
| Omitting Velázquez-López L | -0.03 | -0.28 to 0.22 | 0 |
| Omitting IGENOI study | -0.20 | -0.55 to 0.15 | 42.7 |
| Omitting Yurtdas G | -0.15 | -0.48 to 0.19 | 45.6 |

**Abbreviations:** SMD:standardized mean difference

**Table S16.** Sensitivity analyses for triglycerides excluding one by one each randomized controlled trial.

| **Study** | **SMD** | **95% Confidence Intervals** | **I^2^ (%)** |
| --- | --- | --- | --- |
| Reference | -0.17 | -0.50 to 0.16 | 35.9 |
| Omitting Akbulut UE | -0.20 | -0.68 to 0.28 | 56.7 |
| Omitting Asoudeh F | -0.24 | -0.70 to 0.21 | 49.5 |
| Omitting Velázquez-López L | -0.02 | -0.31 to 0.28 | 0 |
| Omitting Yurtdas G | -0.24 | -0.66 to 0.17 | 48.6 |

**Abbreviations:** SMD:standardized mean difference

**Table S17.** Sensitivity analyses for low density lipoprotein-concentration (LDL-C) excluding one by one each randomized controlled trial.

| **Study** | **SMD** | **95% Confidence Intervals** | **I^2^ (%)** |
| --- | --- | --- | --- |
| Reference | -0.12 | -0.62 to 0.38 | 71 |
| Omitting Akbulut UE | -0.29 | -0.84 to 0.26 | 67.2 |
| Omitting Asoudeh F | -0.20 | -0.91 to 0.51 | 79.1 |
| Omitting Velázquez-López L | 0.12 | -0.18 to 0.43 | 3.5 |
| Omitting Yurtdas G | -0.10 | -0.78 to 0.58 | 80.3 |

**Abbreviations:** SMD:standardized mean difference

**Table S18.** Sensitivity analyses for high density lipoprotein-concentration (HDL-C) excluding one by one each randomized controlled trial.

| **Study** | **SMD** | **95% Confidence Intervals** | **I^2^ (%)** |
| --- | --- | --- | --- |
| Reference | 0.41 | -0.18 to 0.99 | 78.4 |
| Omitting Akbulut UE | 0.46 | -0.37 to 1.28 | 84.8 |
| Omitting Asoudeh F | 0.22 | -0.44 to 0.88 | 76.1 |
| Omitting Velázquez-López L | 0.27 | -0.47 to 1.02 | 83.1 |
| Omitting Yurtdas G | 0.66 | 0.23 to 1.09 | 49.9 |

**Abbreviations:** SMD:standardized mean difference

**Table S19.** Grading of Recommendations, Assessment, Development, and Evaluations (GRADE).

| **Outcome** | **N of participants Intervention** | **N of participants Control** | **Summary effect estimate (SMD) and 95% CIs** | **N of studies** | **Study design** | **Risk of bias** | **Inconsistency** | **Indirectness** | **Imprecision** | **Publication bias** | **Certainty assessment** |
| --- | --- | --- | --- | --- | --- | --- | --- | --- | --- | --- | --- |
| Systolic BP | 140 | 86 | -0.48  (-0.87 to -0.09) | 3 | RCT | Serious^1^ | Not serious | Not serious | Serious^4^ | NA | ⨁⨁◯◯ Low |
| Diastolic BP | 140 | 86 | -0.10  (-0.58 to 0.38) | 3 | RCT | Serious^1^ | Serious^2^ | Not serious | Serious^4^ | NA | ⨁⨁◯◯ Low |
| Weight | 192 | 138 | -0.21  (-0.43 to 0.02) | 5 | RCT | Serious^1^ | Not serious | Not serious | Serious^4^ | NA | ⨁⨁◯◯ Low |
| BMI | 192 | 138 | -0.26  (-0.49 to -0.03) | 5 | RCT | Serious^1^ | Not serious | Not serious | Serious^4^ | NA | ⨁⨁◯◯ Low |
| WC | 81 | 82 | -0.28  (-0.68 to 0.11) | 3 | RCT | Serious^1^ | Serious^2^ | Not serious | Serious^4^ | NA | ⨁◯◯◯ Very low |
| Glucose | 192 | 138 | -0.29  (-0.89 to 0.30) | 5 | RCT | Serious^1^ | Very serious^3^ | Not serious | Serious^4^ | NA | ⨁◯◯◯ Very low |
| Insulin | 168 | 113 | -0.07  (-0.35 to 0.21) | 4 | RCT | Serious^1^ | Not serious | Not serious | Serious^4^ | NA | ⨁⨁◯◯ Low |
| HOMA-IR | 87 | 87 | -0.31  (-0.71 to 0.09) | 3 | RCT | Serious^1^ | Serious^2^ | Not serious | Serious^4^ | NA | ⨁◯◯◯ Very low |
| TC | 192 | 138 | -0.15  (-0.42 to 0.12) | 5 | RCT | Serious^1^ | Serious^2^ | Not serious | Serious^4^ | NA | ⨁◯◯◯ Very low |
| TRGs | 111 | 112 | -0.17  (-0.50 to 0.16) | 4 | RCT | Serious^1^ | Serious^2^ | Not serious | Serious^4^ | NA | ⨁◯◯◯ Very low |
| LDL-C | 111 | 112 | -0.12  (-0.62 to 0.38) | 4 | RCT | Serious^1^ | Serious^2^ | Not serious | Serious^4^ | NA | ⨁◯◯◯ Very low |
| HDL-C | 111 | 112 | 0.41  (-0.18 to 0.99) | 4 | RCT | Serious^1^ | Very serious^3^ | Not serious | Serious^4^ | NA | ⨁◯◯◯ Very low |

**Abbreviations:** BMI: body mass index, BP:blood pressure, CI: confidence intervals, HDL-C:high density lipoprotein-concentration, HOMA-IR: homeostatic model assessment for insulin resistance, LDL-C:low density lipoprotein-concentration, N:Number, NA: not applicable, RCT: randomized controlled trial, SMD:standardized mean difference,TC: total cholesterol, TRGs:triglycerides, WC: waist circumference

^1^Downgraded by one level for risk of bias. All studies judged to have some concerns.

^2^Downgraded by one level for inconsistency. Forest plot shows some inconsistency of the effect estimates between studies.

^3^Downgraded by two levels of inconsistency due to high I^2^ value (I^2^>75%).

^4^Downgraded by one level for imprecision due to small number of participants.
